# Impact of Direct Soil Exposures from Airborne Dust and Geophagy on Human Health

**DOI:** 10.3390/ijerph7031205

**Published:** 2010-03-19

**Authors:** David Sing, Charles F Sing

**Affiliations:** Department of Human Genetics, University of Michigan Medical School, 1241 E. Catherine Street, 5928 Buhl Building, Ann Arbor, MI 48109-5618, USA; E-Mail: dsing@umich.edu

**Keywords:** soil, dust, geophagy, microbiome, genetics, epigenetics

## Abstract

Over evolutionary time humans have developed a complex biological relationship with soils. Here we describe modes of soil exposure and their biological implications. We consider two types of soil exposure, the first being the continuous exposure to airborne soil, and the second being dietary ingestion of soils, or geophagy. It may be assumed that airborne dust and ingestion of soil have influenced the evolution of particular DNA sequences which control biological systems that enable individual organisms to take advantage of, adapt to and/or protect against exposures to soil materials. We review the potential for soil exposure as an environmental source of epigenetic signals which may influence the function of our genome in determining health and disease.

## 1. Introduction

As with all terrestrial organisms, through evolutionary time humans have developed a complex biological relationship with soils. In this paper we discuss two types of direct soil exposure, the first being the continuous environmental exposure caused by airborne soil elements (dust), and the second being the willful (or accidental) dietary ingestion of soil, or geophagy. Endogenous components of soil include minerogenic colloidal clays and trace elements, biogenic organic materials, and biota, any of which may be beneficial, detrimental or toxic, depending on their relative concentrations and the exposure pathway. Soils can also contain human-produced (anthropogenic) pollutants that are a consequence of agricultural and industrial activities. Environmental climate-driven exposure is ubiquitous, as soil is continuously being made airborne and then transported through the atmosphere by the global mechanisms of weather and climate. This deflated soil, or dust, can sometimes be transported and deposited great distances from the point of origin. In this way, humans are now and have been constantly exposed throughout their evolutionary history to deflated soil components, from both local and regional sources and potentially from almost anywhere on the planet.

Geophagy is more localized and is influenced by cultural practices and traditions. The dietary practice of geophagy still occurs in many parts of the world. Geophagy in early humans may have developed as it has with other animals, as a way to consume nutritive minerals and elements otherwise lacking in their usual diet and as a way to detoxify certain plant materials to make them more digestible and nutritious [1,2]. Contemporary dietary geophagy is an active reflection of our evolutionary history, especially with regard to the development of dietary supplements and medicines. We review here the evidence that direct exposure to soil, regardless of the exposure pathway, is an important consideration in understanding the biology of human health and the emergence of abnormal medical conditions.

Our focus on the connection between soil and human health is part of a broader cultural concern about the contribution of the environment to determining the risk of disease [3]. The concern that exposure to soil influences human health is reflected in the efforts of many first-world people to avoid contact with outdoor environments and to maintain a hyper-hygienic indoor environment in an attempt to greatly reduce the amount of potential exposure to disease-causing agents. However, the Hygiene Hypothesis generally states that infections and relatively un-hygienic behaviors, especially during early childhood development, may confer protection against allergic illnesses later in life [4,5]. The Hygiene Hypothesis suggests that children raised in hyper-hygienic environments are often more susceptible to atopic disorders as adults. It is supported by research which documents that young children who are exposed to unhygienic environments, such as those associated with farming and outdoor settings in general, are less likely to develop atopic disorders later in adulthood [4,6].

The field of Medical Geology explores the relationship between human health and exposures to naturally occurring earth materials. Medical geology applies a broad-based, multidisciplinary approach to the health significance of exposures to the multitude of organic and inorganic materials that occur in trace amounts in our environment [7]. With the spirit of medical geology in mind, in this paper we will call attention to evidence that direct contact with minerogenic, biogenic and anthropogenic soil constituents plays an important role in determining the impact of the terrestrial environment on human health. We will suggest that recent advances in characterizing the molecular biology of the human genome reveals heretofore poorly understood biological processes that are involved in determining the genetic basis of variation in response to these environmental agents. In doing so we will call to attention the emerging field of biological research, called epigenetics, which is devoted to understanding how the environment directly modifies the function of the information coded in our genome. We are motivated by our desire to bring into focus three general questions; (1) what is the evidence that human exposure to airborne soil materials contributes to determining human health and the risk of developing disease? (2) What evidence exists which shows that geophagy contributes to determining human health and the risk of developing disease? And, (3) what is the role of variation in genomic and epigenomic factors among individuals in contemporary human populations in determining inter-individual variability in responses to direct exposures to the minerogenic, biogenic and anthropogenic constituents of the soil?

### What Is Soil?

Soil is a complex system of air, water, minerals, organic matter and biota that covers the terrestrial earth in layers above the underlying bedrock [8]. The multitude of interactions among the myriad constituents of soil have been interpreted to infer that soil is a highly dynamic, ecologically complex and diverse living entity [9]. Five important factors in the development of soil are climate, living organisms, parent bedrock, topography and time [10]. Soil is formed as the result of biological and climatologic interactions with the earth’s bedrock. More specifically, the acidic by-products of biological functions and the effects of weathering (wind, precipitation, *etc.*) work over time to slowly break down bedrock materials into smaller and smaller minerogenic constituents. Inorganic elements and biogenic organic materials essential for plant growth gradually accumulate on the surface of these resultant materials to form soils. Fully-developed productive organic soil contains dense and highly diverse communities of macro- and microorganisms. These soil biota are involved in the processing of plant and animal materials which contribute to the development and maintenance of organic soil types.

The physical structure of soils relative to depth from the surface defines the soil profile, which is divided into soil horizons (layers) described generally as A, B and C. Most soils have aspects of all three horizons, although the relative thickness and composition of the horizons may differ greatly from one region to the next [10,11]. Horizon C generally refers to the bedrock, which is the parent material for the top two horizons. The C horizon is the source of the minerogenic materials in all layers of a developed soil. The topmost surface of a soil is described as the Solum, or topsoil. It includes horizon A (the topmost layer) and horizon B (the layer below horizon A). Humans most readily come into contact with horizon A as it supports most terrestrial ecosystems, sustains most human agricultural practices and is most readily exposed to the atmosphere. In horizon A the mixture of biota, organic materials, and broken-down minerogenic particles combine to provide the conditions of optimal fertility and soil structure necessary for supporting the growth of terrestrial plant life, including agricultural production of healthy plant parts for direct human consumption and for animal forage and feed [11].

Horizon B has a higher ratio of minerogenic materials relative to biogenic materials. Materials that form clay are in their highest concentrations in the B horizon. Minerogenic clay materials and organic material accumulate in the B horizon by a process of illuviation (the gradual downward filtering of materials through the soil as a result of gravity). Horizon B only emerges after horizon A has become long established. Tallgrass prairies are the most prolific at producing well-developed horizon A soils. Some undisturbed tallgrass prairie ecosystems have an A horizon that can be up to 5 meters deep [10,12].

## 2. Airborne Soil Constituents and Human Health

**Figure 1.**
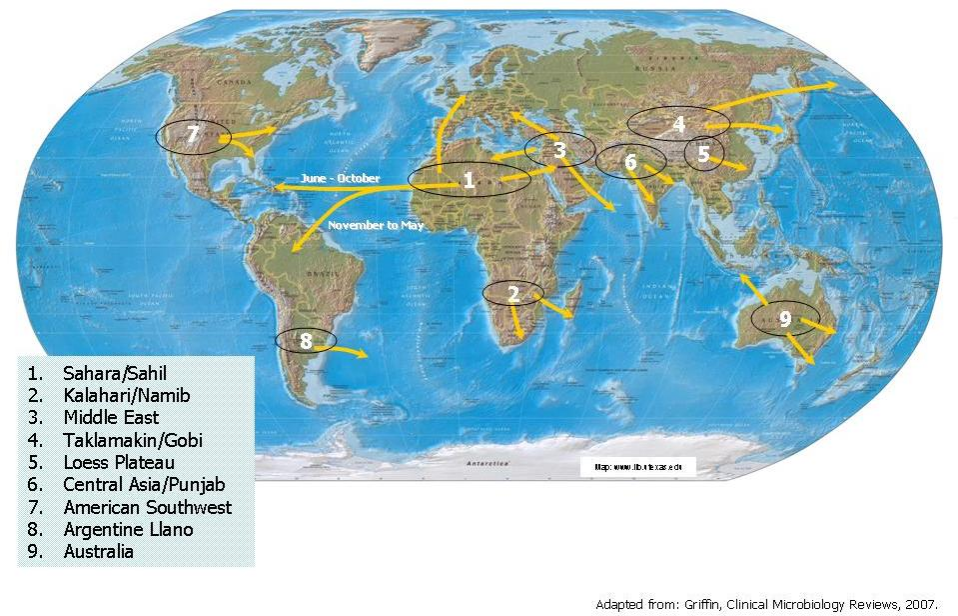
Global climactic systems of dust distribution: sources and trajectories.

A feature of global weather and climate is the constant introduction of surface soil materials into the air. As soils dry, small particles of soil become light enough to be aerosoled and lifted from the surface by wind or physical disturbance. Technically, the process of areal lifting of soil materials is called deflation, and these very small aerosoled soil particles are referred to as dust. Airborne dust includes: minerogenic particles, a collection of colloidal clays and trace elements; biogenic particles, which have organic origins; and viable biota. If windy weather conditions persist over an arid region of the earth’s surface, large amounts of aerosoled soil materials in the form of dust can be deflated throughout all layers of the atmosphere and can potentially be transported great distances before returning to the surface. Climate-driven mechanisms of airborne dust transport exist across the globe wherever deserts and other arid regions lie upwind from more temperate regions [13,14]. Figure 1 summarizes the relationship between the arid regions of the world and the intercontinental rivers of dust that flow throughout the earth’s atmosphere.

Humans are regularly exposed to dust that has been locally deflated, as well as dust carried long distances through the atmosphere after deflation in distant locales. A regular feature of the climate of the Northern hemisphere is the periodic transportation of massive amounts of airborne dust, from the desert areas of Central Asia to Eastern and Western Asia and Europe, from the desert and arid regions of Western North America to places East, and from the Sahara/Sahil expanse of North Africa to Europe and across the Atlantic ocean to the tropical islands and forests of the Caribbean and South America. Dust from the Taklimakan and Gobi deserts, and from the large area of ancient loess deposits in Western China, regularly create large dust storms that persist downwind over the larger cities of Eastern China. These dust storms often last days and sometimes weeks at a time. At tropical latitudes adjacent to the Atlantic Ocean global Easterly wind patterns persist, with weather systems passing off the West coast of Africa and out over the Atlantic Ocean. Stronger, more active weather systems can transport these “packages” of dust-laden African air directly to the Americas. During these “African dust events”, the amount of airborne dust deposited on Caribbean islands increases to 2 to 3 times that of airborne dust collected on “clear atmosphere” days [15]. Studies have estimated that globally as much as 2 billion metric tons of deflated dust are lifted into the atmosphere every year, of which as much as 1 billion tons originate from African soils [13,16]. Presently the effects of climate change, along with a marked increase in agricultural activity in semi-arid regions around the world, are exposing the atmosphere to an increasing amount of deflated soil materials. Perhaps the most conspicuous recent example is the rapid desertification of the Sahel region of Central Africa, resulting from increased human agricultural activity and decreased precipitation associated with climate change. Desertification across this continent-wide swath of Africa has increased the size of the Sahara desert, creating a vast region from which surface soils may become desiccated, deflated and transported across the Atlantic to the Americas.

Research on the impact of this climate-transported African dust reveals an impressive array of environmental and ecological connections between the desert soils of Africa and the myriad ecosystems of the Americas. Direct connections have been made between the ecological health of the Amazon rain forests and the regular deposition of airborne African dust. Studies have shown that the primary source for certain trace minerals crucial to the Amazonian rain forest is not the soils of the Amazon, but rather the periodic deposition of North African soil materials associated with Atlantic-crossing, Saharan dust-laden weather systems [14]. This is one example of how global systems of climate and weather are directly involved in large, complex, and interrelated global systems of climate, geology and biology. Our further understanding of how one large ecosystem (Amazon rain forest) is dependent on another distant ecosystem (Sahara/Sahil desert) via the atmosphere could help to expand our ecological perspective on these complex global systems, and how these global systems impact local human health [14].

## 3. Health Consequences of Airborne Soil Constituents

Dramatic phenomena such as the African dust events and the extended dust storms that regularly plague Eastern China create an increase in human health problems that are well documented in the scientific literature [17]. While such periodic events increase the density of airborne dust, the process of dust transportation and deposition is continuous and occurs even during ”clear” weather. For the time being, there is no scientific evidence for a health benefit from the long term continuous environmental exposure to such low level concentrations of airborne dust. To support the claim that health benefits may arise from dust exposure, it has been suggested that the urbanization of human populations and a cultural preference for an antiseptic microenvironment have, for many people, created a physical disconnect with soils that may be at least partially responsible for a marked increase in asthma and other atopic diseases in Western cultures (the Hygiene Hypothesis) [4,18]. We present here illustrative examples that document the impact on human health of increases in minerogenic, biogenic and anthropogenic constituents of atmospheric dust associated with dust events.

### 3.1. Minerogenic

Minerogenic dust particles small enough to escape the filtering mechanisms of the paranasal sinuses enter the lungs, eventually being deposited in the pulmonary alveoli. Many of the small particulates that settle in the lungs appear to have no negative effect. However, other particulates effectively irritate lung tissue, initializing the formation of fibroblastic cells, which facilitates the formation of collagen and so leads to a variety of pulmonary disorders [19].

A majority of deflated materials in dust events that originate in the deserts of the world are particles of aerosoled free silica (SiO_2_). Silica makes up approximately 60% of the dust deflated in desert regions. Hydrated silica (SiO_2_·nH_2_O) is an essential component of many plant and animal cells including connective tissue, bones, teeth, skin and eyes of humans. This biogenic silica is most common in Saharan dust and is derived from the shells of diatoms and phytoliths. Such biological sources of silica are essentially benign in terms of human health. In Asia and the Middle East much of the aerosoled free silica originates from mineral quartz (minerogenic). Once deposited within the pulmonary alveoli, these quartz-based minerogenic silica particles initiate a fibrotic wound response that can eventually lead to silicosis, a disabling pulmonary condition. Severe silicosis can further damage the immune system by disrupting the ability of macrophages within the lungs to inhibit growth of pathogenic organisms found in airborne dust, leading to an array of bacterial infections. Residents of the Middle East and Central Asia describe these medical conditions generally as “desert lung syndrome”. Autopsies of ancient Egyptian mummies discovered evidence of varying degrees of silicosis within the preserved lung tissue [20]. Long-term exposure to minerogenic dust causing silicosis can eventually lead to non-occupational (re: naturally occurring) pulmonary tuberculosis. While not generally thought of as a disease of arid climates, non-occupational pulmonary tuberculosis has been found to be 25% higher in residents of the Thar desert of Rajastan in India than in residents of the non-desert regions of the same Indian state [19].

### 3.2. Biogenic

An important feature of aerosoled dust is the presence of a large variety of microorganisms, including bacteria and the spores of fungi, which originally inhabit the source soil. Soil contains anywhere between 0 to 10^9^ individual prokaryotes per gram [21]. These viable particles can potentially deflate into the air independently, or adhere to deflated soil by means of adsorption. Most deflated microorganisms travel only short or medium distances before falling back to the ground. However, protected within the niches of individual motes of aerosoled dust, some bacteria and fungi spores that originate in terrestrial soils can survive the cold, extreme aridity and exposure to ultraviolet radiation of high altitude as they are transported around the atmosphere to be deposited far from their place of origin. Dust storms from many different regions of the world have been analyzed, using various methods, for their deflated biogenic components. Table 1 summarizes the total number of culturable bacterial and fungal Colony-Forming Units (CFU’s) detected in various dust storms from around the globe [21]. It should be noted that culturable bacteria constitute less than 1% of species present in the environment.

The relationships between these dust components and human health are difficult to study and confounded with a multitude of other dust-borne agents that may be involved in determining human disease. In one exceptionally well-documented case the short-distance areal transport of a dust-borne pathogen has been associated with bacterial infection, *Neisseria meningtidis*. In North Africa, epidemiologists have identified a so-called “meningitis belt”, where dust storms cause seasonal outbreaks of *Neisseria meningtidis* infections [22]. The abrasive effect of inhaling minerogenic dust upon the nasopharyngeal mucosa creates a favorable environment for infection of *N. meningtidis*. Studies of samples of aerosoled materials collected during dust events on the African continent have detected a host of human pathogens, including *Acinetobacter calcoaceticus*, *Kocuria rosea* and others. However, to our knowledge no studies have linked the African dust events harboring these particular (non-*N. meningtidis*) bacteria to local outbreaks of human disease.

In the North American Southwest an endemic disease, Valley Fever, or Coccidioidomycosis, is caused by exposure to spores of different species of a single genus of an indigenous soil fungus (*Coccidioides spp*.). Fungi of the *Coccidioides* genus prefer light sand or silty soils common to arid and semi-arid regions of the Southwestern U.S. states. Outbreaks of the disease are dependant on seasonal climactic variations in temperature and precipitation. In its saprophytic or mold phase *Coccidioides spp.* live as mycelia embedded in the soil. Saprophytic *Coccidioides spp*. are among the hardiest organisms found on the planet. Once established, *Coccidioides spp*. can progress through their life cycle into the parasitic stage, whereby the organism produces large amounts of arthrospores. Arthrospores are spherule cells of the organism, each of which contain endospores that can individually propagate into a new spherule cell. If climactic conditions create long periods of dry and windy weather the arthrospores of *Coccidioides spp*. become deflated along with other soil constituents. Once airborne these arthrospores can be inhaled by humans. After inhalation, arthrospores of *Coccidioides spp.* begin to multiply rapidly within the lungs of infected humans. Most people infected with the arthrospores of *Coccidioides spp*. (~60%) present no symptoms or only mild, cold-like symptoms, while up to 40% of those infected develop flu-like symptoms within three weeks of infection. These flu-like symptoms are described as Valley Fever. Those individuals whose infection emerges as Valley Fever suffer through a one to three week period of flu-like symptoms, including respiratory distress and fatigue, which is the most common symptom. Approximately one percent of those who present with Valley Fever develop a more advanced form of the disease, Coccidioidal Meningitis, whereby the infection spreads beyond the lungs into other organs of the body, including the skin and skeletal structures. Coccidioidal Meningitis is fatal in nearly 100% of those individuals who develop this highly advanced form of the infection [23].

### 3.3. Antropogenic

The negative health effects of exposures to aerosoled anthropogenic particulate materials, or air pollution, are well-documented. Air particulates of anthropogenic origins have been connected with a wide array of human pathologies mostly associated with cardiovascular and pulmonary diseases [24]. In many regions of the world the health effects of dust originating from the soil are greatly exacerbated by anthropogenic-originating airborne pollutants. The oldest form of airborne anthropogenic particulate materials is dark carbon from coal, wood and/or dung fires. Today in Africa and Asia up to a billion people still use coal, wood and dung fires as their daily primary source of heat for cooking. These cooking fires, which burn almost continuously in some regions, contribute massive amounts of dark carbon soot into the atmospheric mix of deflated soil materials and microorganisms. In areas of dense air pollution carbonaceous materials make up between 20% and 50% of airborne particulate mass [24,25].

## 4. Willful Ingestion of Soil: Geophagy

Most primate species are geophageous. Archeological evidence suggests that *Homo erectus* and other early hominids included geophagy in their dietary routines [26,27]. Contemporary geophagy involves the collection, preparation and dietary consumption of specific soils for their nutritive or medicinal value. Unlike airborne soil exposures, which are ubiquitous and affect every living person to some degree on a continuous basis, geophagy can be a willful behavior, often predicated on cultural beliefs and dietary practices. Since pre-history, humans have consumed certain colloidal mineral- and trace element-rich soils as a supplement to their otherwise nutrient-poor local diet, have used certain soils as detoxifying agents necessary for making certain food products edible, and have used selected soils for medicinal purposes, usually as treatments for gastrointestinal ailments. Soils selected for human dietary and medicinal geophagy are usually clays, which can possess adsorbing qualities that aid in removing toxins from plant products, and which have a higher concentration of trace elements and colloidal minerals than other soil types.

### 4.1. Who Eats Soil, and Why?

Medicinal soils (or medicinal earths) are willfully consumed or applied topically for treatment of any number of human disorders. In the first-world commercial products containing medicinal, colloidal clays are sold over the counter. These include vitamin and mineral supplement tablets and antacids (Kaopectate, Pepto Bismol, *etc.*). Accidental geophagy occurs when people eat improperly washed fresh vegetables, or vegetables whose skin has become damaged during growth or harvest. Accidental geophagy can have deleterious health effects if soil that contains pathogenic materials (such as fertilizers, pesticides and/or pathogenic organisms) becomes adhered, or introduced internally through injury, to plant parts consumed as food.

It is notable that most willful geophageous behavior is culturally driven, and that clinical evidence of beneficial geophagy is not generally understood by cultures that regularly engage in geophagy. Instead, the beneficial aspects of nutritive and medicinal geophagy have developed anecdotally in geophageous cultures as a consequence of many generations of local traditions that include a close interaction with regional ecosystems and environments. Presently, direct ingestion of soil is not a common dietary practice in most of the first-world where such behavior is considered by some to be a psychological condition known as Pica (the pathological ingestion of non-food materials, such as glass, metals, wood, *etc.*) [28]. Colloidal mineral and trace element dietary requirements are usually met in the first-world by the consumption of more readily available fresh fruits and vegetables, by eating processed food products that have been artificially enhanced with nutritive minerals, vitamins and trace elements, or by consuming manufactured vitamin and mineral supplements in the form of pills or capsules.

In much of the remaining world, including regions of Africa, Asia, South and Latin America, the Pacific Islands and particular areas of the American South, geophagy remains a common practice. People from each of these specific regional cultures have their own particular methods and practices that vary according to local soil types and cultural motifs of behavior. Despite this cultural heterogeneity, a few generalizations can be made about who eats soils and why. In many places geophagy is practiced by pregnant women who eat mineral and trace element rich soils as a prenatal dietary supplement. For many others, geophagy is included in the daily routine as a nutritive supplement and as a way to curb hunger. For still others, geophagy is a dietary method of last resort, and certain soils are prepared and eaten as starvation food [29,30].

In general, geophageous soils are most commonly collected from exposed banks of rivers or adjacent to fresh-water seeps and springs. These soil licks and clay pits may have been initially discovered by watching local indigenous animals who seek the soils for their own specific geophageous dietary needs. Some of these soil collection sites have been used by humans for many generations, and in some instances, for thousands of years. A common practice among modern geophageous peoples is to identify and collect certain clays, mix them with available animal fats and/or grain flours, mold them into serviceable portions, and then bake them or allow them to dry naturally. Pre-made soil cakes can be found at local marketplaces throughout Africa, Asia and Latin America, where the prepared soils are purchased by market-goers as a part of their food shopping [29,31].

Until the adaptation of fire all foods collected for consumption by humans were eaten raw. The majority of food collected was meat from animals, as most raw plant materials were found to be non-palatable or toxic to humans. Perhaps the most important result of geophageous behavior in pre-historic peoples was the discovery that mixing certain clays with certain plant products made the plant products more palatable. The classic example is that of the domestication of the potato (*Solanum spp*.). Archaic potato varieties are known to have high concentrations of glycoalkyloids which can be toxic and even fatal to humans. It has been suggested by Johns that geophageous knowledge gained from consuming clays inspired native peoples of Latin America to begin cultivating the otherwise toxic potato varieties directly in soils that contained the clays used otherwise for geophagy [32]. By cultivating potatoes in clay soils with good adsorption qualities, toxic glycoalkaloids are effectively reduced in the final food product. Adoption of this cultural practice allowed for an expansion of the cultivation of potato as a food staple and may have been a factor in the agricultural evolution of prehistoric cultures [33]. Presently, the Pima Indians of the American Southwest regularly use geophageous clays in an acorn-based dough that is baked and eaten. The otherwise bitter and mildly toxic meats of some Oak (*Quercus spp*.) acorns are made more palatable and digestible by mixing with certain locally-found colloidal clays. Geophagy is also a developmental behavior for young children, who consume non-food materials as a means of exploring and assessing their environment [29,33].

### 4.2. Health Consequences of Geophagy

Most of the recent literature on the health consequences of geophagy focuses on either the deleterious effects of eating soils in the general population, or on the efficacy of including geophagy in the diet of pregnant women. The negative health consequences of geophagy are well-documented [34]. These include: toxic reactions to soils contaminated with lead (Pb) or with anthrogenic (human-introduced) pollutants; the ingestion of soil high in potassium (K), which leads to hyperkalemia; the ingestion of endoparasitic organisms, such as geohelminths (helminth nematodes, r.e.: hookworm, *etc.*) and *Clostridium tetnai* (cause of tetanus); chronic intestinal blockage; and excessive tooth wear [34–36].

Generalizations can be made about the potential health benefits of geophagy. Colloidal clays, such as the kaolinitic clays, are consumed for their anti-diarrheal properties. This geophageous practice is mimicked in the developed-world by the use of commercially available colloidal clay products such as Kaopectate© among others [33]. The adsorbent qualities of colloidal clays also act as detoxifying agents when combined with plant foods that are high in glycoalkyloids and other toxic materials. Geophagy is also practiced as a nutritive supplement to normal regional diets where certain minerals are otherwise lacking in the more conventional plant and animal food sources. Certain geophageous soils can contain usable amounts of nutritive minerals; most clinical studies on this geophageous activity report that some soils supply high levels of calcium, which is otherwise in low supply in the regional diets studied [1,38].

The most commonly reported incidence of nutritive geophagy in modern scientific literature focuses on geophagy in pregnancy [33]. In some cultures an increase in geophageous behavior among young women is considered diagnostic for pregnancy [30]. Regardless of cultural motifs associated with the behavior, geophagy during pregnancy can produce nutritive and medicinal benefits. In the first-trimester, certain colloidal clays are consumed for their anti-nausea properties. They have been shown to be highly effective in reducing the symptoms of morning sickness [31]. As a woman’s pregnancy progresses into the second- and third-trimester geophagy is a way to obtain important minerals critical to pre-natal development of the fetus. Perhaps the most notable component of pregnancy-related geophagy is the ingestion of calcium-laden soils. Calcium is important to the development of fetal skeletal structures and may also reduce the risk of pregnancy-induced hypertension. The geophageous intake of calcium-rich soils is especially important in certain regions of Africa which have a serious lack of calcium available in their food diets [39,40].

Another aspect of geophagy that has proven to have a direct impact on the risk of developing atopic disease is the relationship between humans and infestations of endoparasitic organisms such as the geohelmith worms. Infestations of helmith worms are associated with poor hygiene and can lead to schistosomiasis and other geohelminth-related diseases. The positive influence of geohelmith parasitic infestations on the human immunological response to allergens is well-documented. Epidemiological studies of the helminth-human relationship have revealed that infected tissues are induced to produce higher levels of T-cell cytokines, which confer a certain amount of protection against atopic disease in infected individuals. These cytokines include the interleukins IL-4, IL-5, IL-10 and IgE. These relationships have been aggressively suppressed by modern medicine in the last century, as the negative health consequences of geohelminth worm infestations far outweigh any potential positive aspects [36].

## 5. The Biological Basis of the Relationship between Soil and Human Health

*Soil is the interface between lifeless cosmic rock and all terrestrial life and it is the fundamental source of life* [37].

Soil, the layer of minerals, living microorganisms and dead plants and animals blanketing the planet, is the mother of all terrestrial life. Over evolutionary time all forms of life have developed biological systems that enable individual organisms to take advantage of, adapt to and/or protect against exposures to materials that originate in the soil. These biological systems involve anatomical structures and metabolic pathways that facilitate the extraction of nutrients from food and water that are essential for growth, development and the maintenance of a healthy body. The human immune system has developed in parallel with these life-supporting processes to protect against adverse effects of life threatening components of soil, water and food.

The immune system is the result of nearly 3 million years of natural selection for genetic variations that produce the antibodies that protect life from adverse effects of exposures to environmental agents that have the potential to threaten the integrity of biological processes that support life. The inherited biological abilities of the immune system to isolate the human organism from pathogenic invaders that have their origins in the soil is essential for avoiding disease. The water we drink, the food we eat and the air we breathe, regardless of geographic location, can include minerogenic, biogenic and anthropogenic components of the soil that can act as antigens. The NIH National Institute of Allergy and Infectious Diseases has reported that airborne dust is the primary source of environmental agents that foster human allergic stress [41]. The biological basis of a healthy response of the human immune system to airborne dust has developed as a consequence of long-term exposures, on an evolutionary time scale to minerogenic, biogenic and anthropogenic components that are ubiquitous components of the ecological history of humans. An inherited hypersensitivity of the immune system to respond to a particular ubiquitous airborne antigen or the exposure to an unfamiliar aerosoled soil material may trigger responses of the immune system that can result in asthma and other related conditions.

The surfaces of the healthy human body, both external (skin) and internal (sinuses and lungs, the alimentary canal), are populated with a plethora of species of microorganisms. The total number of microbial cells that reside in or on a healthy human (the microbiome) outnumber the body’s own cell count by at least an order of magnitude. Some of these microorganisms assist the immune system in protecting the body from pathogenic microorganisms. Many are essential inhabitants of the gut necessary for extracting nutrients from food.

The microorganisms found on human internal and external surfaces are also commonly found in soil [42–44]. As humans are born basically sterile of microorganisms, with no gut microbiota present, the microbiome is initiated as a consequence of environmental exposures beginning very early in life (e.g., from mother at parturition, mother’s milk and hand to mouth transfer). The human microbiome is further populated in any one of three additional ways: (1) Deflated soil materials which float through the air and come to rest on the skin or are breathed into the pulmonary system. The action of simply being active outdoors can trigger such exposures; (2) Microorganisms associated with soil can be ingested, either by direct, willful geophagy or by eating fruits and vegetables that are not completely clean of soil residues (accidental geophagy); and (3) Some common gut bacteria can be purchased in tablet, capsule or powder form, or in processed foods such as certain yogurt products, and ingested as probiotic dietary supplements. As a consequence of a deeper understanding of the microorganism-human commensal relationship, researchers have come to describe the human body as a super-organism. The host human and the communities of resident microorganisms are each dependent on each other for their survival [45].

The total amount of genetic information represented in the microbiome exceeds the amount contained in the nucleotide base sequence of the average human genome by approximately 100 times. The evolutionary roots of the human microbiome trace back to the soil [45]. Recent technical advances in DNA sequencing have made it feasible to study the genomes of microorganisms that make up the microbiome. Such metagenomic studies use this information to isolate and characterize the phylogenic relationships among the bacterial species that make up the microbiome. A fraction of the genomic information found in the species of the microbiome is shared by all contemporary populations of the suite of human gut microorganisms and their counterparts in soil [46,47]. As many as 80% of the species of microorganisms that have been identified in the soil have genomic analogues in the human gut.

We next turn to a brief overview of how contemporary actions of inherited variations and variations among humans in the functions of the inherited genetic elements determined by the environment (epigenesis) can influence the impact of contact with soil on human health. Variation in the impact of soil constituents on an individual’s health is influenced by many genetic variations that regulate complementary components of normal biological processes. We focus on the immune system because it plays a central role. Variation in the epigenetic signals that result from exposures to environmental agents which threaten the normal functions of the human body adjust the human immune response by modifying the expression of the genetic information encoded in the human genome (which in turn influences the immune system).

### 5.1. Inherited Genomic Variations

The DNA sequence of nucleotide bases that makes up the human genome encodes approximately 6 billion bits (three billion from each parent) of inherited information. Most of this information is involved in controlling the growth and development of the individual and the regulating the biological processes that support life. As exposures to soil have been continuous throughout human evolutionary history, it may be assumed that air-borne dust and direct ingestion of soil have influenced the evolution of the DNA sequence that controls the human immune system. In contemporary populations of humans some of this encoded information is fixed, and does not vary from individual to individual. However, a significant fraction of the total genome consists of portions of the DNA sequence which vary among individuals within and between contemporary populations. This is because the very DNA sequence variations that have been selected to protect against pathogenic agents in the environment are susceptible to mutational changes. Some of these mutations will result in damaging, or weakening the competence of, the immune system in ways that will influence one’s immunological response to environmental exposures to minerogenic, biogenic, and/or anthropogenic constituents of the soil. Asthma is a common atopic disorder that has its origins in the failure of the immune system to function properly when exposed to certain airborne constituents [48].

Hundreds of genes have been implicated in determining how the human immune system influences protection from, or pathogenicity of, exposures to specific agents that are known to influence the development of asthma. Progress in indentifying and characterizing the impact of these genes has been enhanced by recent advances in measuring and analyzing genetic effects. In a review, Ober and Hoffjan called to attention variations in 25 genes which have been associated with asthma or atopy phenotypes in six or more independent populations [49]. They reported that DNA sequence variations in a subset of eight of these genes have been associated with an asthma or atopy phenotype in more than 10 independent studies of human populations. As of 2009, the list of candidate susceptibility genes included 43 whose effects have been replicated in two or more studies [50]. Biologically significant effects of the *TNF* gene, located on chromosome 4, have been replicated in studies of seventeen populations.

The onset of asthma at a particular age is consequence of the interactions between the effects of variations in one or more of the many genes that are involved in the regulation of the immune system with the effects of past and present exposures to environmental factors that are unique to the individual. One biological trigger for asthma in humans which has been studied extensively is the inhalation of bacterial endotoxins. Lipopolysaccaride (LPS) is a common bacterial endotoxin. LPS is typically a component of aerosoled indoor dust which includes endotoxic bacteria. Once inhaled the aerosoled dust containing LPS settles within the tissues of the primary airway where it affects the epithelial cells of the airway walls. Humans respond to LPS exposure in different ways, ranging from no reaction to respiratory inflammation and asthma symptoms. It has been suggested that certain mutations in the Toll-like Receptor 4 gene (*TLR4*), in combination with other genes involved in determining the immune response, determine varying degrees of human responsiveness to LPS. Individuals with the common allelic forms of the *TLR4* gene are not generally affected by exposure to environmental dust. However, individuals with one particular mutation of the gene (known as the *Asp299Gly* mutation) are predisposed to respond negatively to LPS-laden dust. In these individuals exposure to LPS-laden dust sets off a chain of metabolic reactions, each of which is regulated by other genes, which can lead to the emergence of symptoms of asthma [51–53].

Contemporary genetic studies of how the human immune system responds to particular components of airborne dust are compromised by the levels of anthropogenic pollutants which permeate the atmosphere at all levels and at all geographic latitudes. Quite simply, there is nowhere on earth to study the genetic basis of the response of the immune system to airborne dust where the air does not contain a mix of human-produced hydrocarbons, radioactive isotopes and other anthropogenic materials that did not exist throughout most of the evolutionary history of our species [13].

### 5.2. Acquired Modifications of the Human Genome; the Epigenome and Epigenetic Variation

Traditional genetic studies of phenotypic variation have sought to identify and characterize the independent roles of variation in the DNA sequence that characterizes the human genome (nature) and variation in exposures to modifying environmental experiences (nurture). Epigenesis is a term used to describe alterations in the function of the genome (which are determined by exposures to environments both internal and external to the organism) that do not involve changes in the DNA sequence. Epigenetic (‘above genetics’) studies seek to identify and describe these heritable (cell to cell and generation to generation) changes in the pattern of gene expression. These changes are determined by methylation of particular sites in the DNA sequence and by modifications of histone proteins (structural components of chromosomes) that are involved in the process of epigenesis. Epigenetic modifications influence the function of the DNA by controlling access to the processes that translate the information coded in the DNA into biologically active molecules.

Epigenetic mechanisms modify the expression of DNA and/or RNA by attaching a methyl group to the DNA (methylation), by removing a methyl group (demethylation) or by modifying the histone proteins that package the genome. Changes in the DNA methylation pattern and modifications of histones are essential in the timing of the control and regulation of genes during normal development. Demethylation of particular gene regions has been implicated in determining abnormal tissue behavior resulting in cancer [54].

During normal development the contribution of the genome to determining tissue and organ differentiation is controlled by changes in the epigenome that are transmitted from one cell generation to the next. Different patterns of epigenetic “marks” are associated with the genomes of cells that form different tissues of the body. Normally this process of epigenesis begins anew each generation. Exceptions involve epigenetic markings that are transmitted in gametes from parents to offspring and grandparents to grandchildren. These exceptions, called imprinting, have been associated with the trans-generational inheritance of a number of human diseases [55,56].

Recent epigenetic research has established that tissue specific patterns of methylation may be triggered by exposures to environments external to the organism that can occur at any time from conception through adulthood. Szyf suggests that epigenetic modifications enable the static human DNA sequence to respond to dynamic changes in the environment throughout the lifetime of the individual [57]. Many phenotypic variations that manifest in adult humans may be in no small part regulated by the history of exposures to external environments during early development [55,57,58]. Seminal experimental mouse studies have established that components of the mother’s diet during pregnancy can influence heritable phenotypes of the adult offspring [55,56,60]. Subsequent studies have established that maternal exposures to particular chemicals and endocrine disrupters can influence the pattern of epigenetic markings in adult offspring [20,55]. Additionally, exposures to different types of maternal care (e.g., grooming behaviors) can trigger different methylation patterns in developing offspring, and thus predispose to different phenotypes in adults [57,61].

The epigenetic response in adult humans to airborne constituents provides further insight into our understanding of the relationship between soil and human health. Several human studies have suggested marked changes in DNA methylation patterns in association with exposures to airborne particulates from anthropogenic sources. In one study of the methylation patterns associated with the 5’ region of the *ACSL3* gene revealed that maternal exposures to certain components of air pollution (specifically polycyclic aromatic hydrocarbons or PAH’s) initiate DNA methylation of the *ACSL3* gene region of fetuses in utero. These methylation patterns have been linked to the development of childhood asthma [62]. The dynamic nature of DNA methylation is documented by a study of blood samples from elderly participants that found significant changes in methylation of DNA of white blood cells as soon as four hours after exposure to anthropogenic particulates commonly found in traffic dust. Further examination of the samples indicated that methylation of DNA decreases within four hours of the exposures, and the demethylation process continued for seven days [58]. At this time no direct connection has been made between these rapid DNA methylation changes due to exposure to traffic dust and human health. It is of concern that such a rapid alteration of the DNA methylation pattern suggests that other exposures having similar rapid effects may be difficult to evaluate medically.

Air particles are also known to initiate the natural production of different reactive oxygen species. This increase, exacerbated by dark carbon, heavy metals and other deflated anthropogenic materials, can increase oxidative DNA damage. Increased oxidation damage can diminish the efficiency of methylation of the DNA, resulting in hypomethylation, or a general reduction in DNA methylation across the entire genome.

In recent years there has been an exponential expansion of our comprehension of metagenomic information carried by the myriad bacterial communities that live in symbiosis with humans. The ecology of the human metagenome is complex, with many genera of bacteria working in symbiosis with each other and with the individual human to regulate various human physiological functions, and to regulate epigenetic responses to environmental exposures. From an epigenetic perspective, this complex relationship has huge implications for human health. It is becoming increasingly apparent that the biological response of individual humans to dynamic changes in the environment throughout one’s lifetime are influenced to no small extent by the interplay between the genomic and epigenomic information of human-hosted bacteria and the genomic and epigenomic information of the human host.

## 6. Conclusions

Soil provides the fundamental basis of all terrestrial life. Our biological makeup has evolved (and is evolving) to accommodate and protect from continuous exposure and re-exposure to biogenic, minerogenic and anthropogenic soil materials. The potential for soil exposure as an environmental source of epigenetic signals which influence the function of our genome in determining health and disease is great. The phylogenic origins and similarities of soil and human gut- and skin-hosted bacteria suggest an ecological continuum between soil and human health.

Research to understand the biological connections between biogenic, minerogenic and anthropogenic materials of the soil and human health should reveal a better understanding of the complex interactive roles of our genome and environmental histories over a lifetime and over the evolutionary history of our species. Such knowledge could be the basis for defining opportunities for individuals to adjust their behaviors to effectively modify their environments in ways that will improve health and prevent disease.

## Figures and Tables

**Table 1.** Concentration of culturable bacteria and fungal spores in dust storms.

		Bacterial CFU m^−3^		Fungal CFU m^−3^	
Sample Site	Dust Source	Background	Dust	Background	Dust
Kansas	Kansas	<10	2,880–42,735	ND	ND
Junction, TX	Texas	<450	>1,544	NA	NA
Mali	Sahara/Sahil	200–1,100	720–15,700	0–130	80–370
Israel	Sahara	79–108	694–995	31–115	205–226
U.S. Virgin Islands	Sahara/Sahil	0–100	90–350	0–60	30–60
Korea	Gobi/Taklamakan	105–1,930	225–8,212	100–8,510	336–6,992

CFU m^−3^ = Colony Forming Units per cubic meter of air

ND—No Data

NA—Not Applicable

Adapted from: Griffin, DW; Clincal Microbiology Reviews, v. 20 (3), 2007
